# Evaluation of Pain in Adults With Childhood-Onset Disabilities and Communication Difficulties

**DOI:** 10.3389/fneur.2021.722971

**Published:** 2021-10-05

**Authors:** Taylor Jersak, Garey Noritz

**Affiliations:** ^1^Department of Internal Medicine, The Ohio State University Wexner Medical Center, Columbus, OH, United States; ^2^Department of Pediatrics, Nationwide Children's Hospital, Columbus, OH, United States

**Keywords:** cerebral palsy, neurologic impairment, childhood-onset disability, adults, pain assessment, palliative care

## Abstract

Adults with childhood-onset disabilities, particularly those with central nervous system impairment, commonly experience pain. Because many such individuals have difficulties in communication, caregivers and medical professionals must identify and interpret non-verbal behaviors as indicators of pain. This process is challenging and can lead to poor outcomes through delayed or incorrect diagnosis and treatment. Most research in the evaluation of pain in individuals with neurologic impairment has focused on the pediatric population, and evidence-based guidelines do not exist for adults. The purpose of this paper is to review current recommendations for pain assessment in adults with communication impairment. This approach includes guidance for history-taking, pharmacologic review, physical examination, and the judicious use of laboratory and imaging tests. Finally, we discuss adult-specific diagnoses to consider when evaluating pain in adults with childhood-onset disabilities and communication difficulties.

## Introduction

Because of advancements in medicine, people with childhood-onset disabilities commonly live into adulthood (1, 2). One of the most common childhood-onset disabilities is cerebral palsy (CP), a group of movement disorders caused by a disturbance to the fetal or infant brain (2, 3). Because cerebral palsy is highly prevalent, with ~2–3 per 1,000 live births affected (3, 4), we will use this condition as an exemplar of childhood-onset disabilities related to neurologic impairment.

Although the brain disorder causing cerebral palsy is not progressive, the manifestations of CP can change over time, and this population experiences numerous physical and psychological challenges, such as pain, that can change and persist into adulthood (2, 5, 6).

In children and young adults with cerebral palsy, prevalence of pain ranges from 14 to 76% (2, 7). More specifically, pain is more common in non-ambulatory adults with cerebral palsy, with a prevalence of 79% (8).

There are several other common childhood-onset disabilities in which pain is commonly experienced (9–11). Spina bifida has a prevalence of pain in young adults ranging from 34 to 40% (9). Additionally, childhood CNS cancer survivors have a prevalence of pain in young adults of 36% (11), and a relative risk of pain of 7.9 compared to unaffected siblings (12).

Despite pain being common in adults with childhood-onset disabilities, it is frequently underrecognized and undertreated (6). Diagnosing the underlying etiology of pain is difficult, particularly in individuals with communication difficulties who are unable to identify and relay the location, severity, or pattern to their pain. Common sources of pain in adults with CP and other CNS disorders include musculoskeletal abnormalities, spasticity, gastrointestinal dysmotility, feeding tube dysfunction, tooth decay, and acute injuries (2, 7, 8). There are no standardized tools recommended for measurement of pain in adults with childhood-onset disabilities or with barriers to communication. Research is limited for both adults and children. We will review the available literature of pain assessment in individuals with communication impairment and then describe an approach for evaluation of pain in adults with childhood-onset disabilities.

## Current Literature

### Pediatric Literature

Children with neurologic impairment commonly experience pain, and pain is notably more common in individuals with dyskinetic and mixed cerebral palsy types, with a prevalence estimate of 85% (13). The frequency with which children with moderate to severe cerebral palsy experience pain is high, with 44% of parents reporting pain in their children “once or twice” to “a few times” over the course of 4 weeks (14).

The evaluation of pain in children with neurologic impairment is also complex due to having multiple potential sources of pain, as well as frequent barriers to communication. Common sources of acute pain in children with cerebral palsy may be directly related to the underlying cerebral palsy (e.g., hip dislocation), secondarily associated with CP (e.g., nephrolithiasis related to immobility), or not related at all (e.g., appendicitis). Acute exacerbations of chronic pain from neuropathic etiologies and spasticity are also important to consider (7, 13–15). The most common locations of pain in children with cerebral palsy are the lower extremities, back, and abdomen, though this population has numerous other locations that should be evaluated as well (7).

Complicating this process is that many children with neurologic impairment are unable to verbally describe the location or characteristics of their pain. Some individuals may also express atypical behaviors to indicate pain, such as altered mental status, self-injurious behaviors, and laughter (14). In these situations, health care providers must rely on history provided by other informants, such as relatives or other caregivers, to evaluate pain and create a differential diagnosis.

There have been numerous assessment tools developed to evaluate pain in children with neurologic impairment and communication difficulties (14–19). Several tools that have been validated for use in the pediatric population, though none of the tools have been proven to be more effective and accurate than the others, and none have been validated in adults (14, 16, 18, 19).

Most recently, the Guidelines for Ruling Out and Assessing Source of Pain (GRASP) was developed as an assessment tool to evaluate pain in children with medical complexity, including cerebral palsy, who are unable to verbally express pain (15). The tool helps to provide a systematic approach to guide clinicians through the history, physical examination, and initial workup of a pain evaluation. The GRASP uses a flowsheet to guide the history and physical exam. If the child is not at their baseline, or there are any concerns in the history and physical exam, the flowsheet provides suggestions for an initial diagnostic workup. The GRASP also includes a broad differential diagnosis, considering common neurologic, infectious, respiratory, gastrointestinal, genitourinary, musculoskeletal, and other etiologies that may cause pain in this population. A mixed-methods study provided initial content and face validation of the tool (15).

### Adult Literature

Children with neurologic impairment now commonly live into adulthood and make up an increasing percentage of the general population. Increasing age is related to higher levels of pain, and, similar to findings in the pediatric population, adults with cerebral palsy who are non-ambulatory experience more frequent pain (8). Importantly, pain is associated with a reported lower quality of life in adults with cerebral palsy, highlighting the need to effectively evaluate and treat their pain (8).

Despite pain being highly prevalent in adults with cerebral palsy, research is limited in this population (2). Most literature on this topic discusses the prevalence and characteristics of pain in adults with cerebral palsy and neurologic impairment. Prevalence of pain in individuals with cerebral palsy is similar between adolescents and adults, suggesting that pain starts early in life and continues, especially as neuromuscular complications change and progress (2). For example, pain in the upper extremities increases as the aging process occurs, possibly due to overuse of arms over time and with increased dependence on mobility devices. The most commonly reported sites of pain in adults with cerebral palsy include the back (most common), lower extremities, and upper extremities. Interestingly, this distribution of pain differs slightly from children, whose most common source of pain is primarily the lower extremities (6). Other common sources of pain to consider include bladder distension or irritation, constipation, kidney stones, gastroesophageal reflux, subluxed or broken bones, and dental caries (3).

Additionally, pain is underrecognized in individuals who cannot verbally communicate or who rely on reports by others (6). This finding is especially concerning, given that pain is prevalent in all patients with CP regardless of the severity of the motor disability (2, 20). Because of the recognition that many people with cerebral palsy have communication barriers, one study compared the pain ratings between individuals with cerebral palsy and observers. Although there was moderate agreement between observers, there were major differences between patient and observer ratings (21). Although pain is an important and prevalent problem in adults with childhood-onset disabilities, there are no tools validated for use in non-verbal adults with neurologic impairment to evaluate pain.

## Discussion

Given the paucity of evidence-based recommendations for evaluation of pain in adults with childhood-onset disabilities, we will describe the approach taken in our program to evaluate pain in this population.

### Take a Systematic History

There are several considerations to obtain a thorough and accurate history. The most important feature is to recognize the value of the history provided by the person's caregivers, as they spend the most time with individual and can notice slight changes in patterns and behavior before most others, especially health care providers. These changes are often the best clues to the diagnosis. We ask about the duration and pattern of the behavior, such as if it is worse before or after meals, before or after stooling, in certain positions, or temporally related to menses.

It is well-known that individuals with medical complexity may express pain using non-verbal signs or atypical expressions (14). Caregivers are familiar with the persons' patterns of verbal and non-verbal communication, and can detect a change. The caregiver may also be able to relay what similar symptoms have meant in the past and we often ask the caregivers what they suspect based on their previous experiences. Often, recurrent urinary infections, kidney stones, or ventricular shunt malfunction may present similarly to previous episodes, what we call the “recapitulation of symptoms”^1^. Asking the caregivers for their diagnostic thoughts also helps us understand their areas of concern. This highlights the importance of developing a longitudinal relationship with individuals and their caregivers.

### Comprehensive Physical Examination

A comprehensive physical examination is the next step in evaluation of pain. Special attention should be paid to the vital signs, joints, teeth, dependent areas, and any medical devices such as a ventricular shunt, implanted pump, tracheostomy, and feeding tube. It is important to relate each physical exam finding back to the person's baseline, as this may not be the same baseline as others.

### Diagnostic Testing

Findings in the history and physical examination should guide the diagnostic testing. The initial workup typically includes a complete blood count with differential, comprehensive metabolic panel, urinalysis, cultures, and inflammatory markers to delineate pain sources. Imaging is also commonly useful to detect specific areas and organs that may be causing pain, including *x*-ray, ultrasound, and sometimes CT scan. Based on results of the initial workup, further testing may be indicated to identify the etiology of pain more specifically. If this first round of testing is unrevealing and symptoms persist, it is often useful to image the abdomen because intra-abdominal pathology is commonly difficult to detect with physical examination.

### Special Considerations for Adults With Childhood-Onset Disabilities and Communication Difficulties

Although it is recognized that having a standardized approach is important to evaluate and diagnose the cause of pain in individuals with communication difficulties, many current assessment tools are validated only for use in children (15). Many diagnoses that lead to pain apply to both children and adults, such as bowel obstruction, kidney stones, and fractures, and the use of a pediatric assessment tool may be helpful to guide initial workup. However, there are several diagnoses that are relatively common in adults and uncommon in children. Table 1 illustrates findings in the history and physical examination that could be possible indicators of pain in adults (1, 15, 22–24). The diagnoses associated with these findings should also be considered in the evaluation of behavior change in adults with childhood-onset disabilities and communication impairment.

**Table 1:** Findings in history and physical examination that may indicate pain as source of behavior change in adults with childhood-onset disabilities and communication impairment.

**Source of pain**	**Common findings in history**	**Physical examination findings**	**Diagnostic considerations**
Neurologic	Altered mental status/acting withdrawn Rapid decline in mobility or change in gait Recurrent vomiting, regurgitating, or feeding refusal New onset incontinence Increase or change in pattern of self-injurious behavior or others signs of pain	Diaphoresis Increased spasticity Increased weakness Signs of increased intracranial pressure (bradycardia, hypertension, irregular breathing, cranial nerve palsies, etc.)	CT/MRI of brain X-ray of spine CT/MRI of spine
Cardiovascular	Increased fatigability Pain or discomfort with exertion Rapid changes in weight	Lower extremity swelling Signs of respiratory distress Orthopnea Tachycardia Hepatomegaly	ECG Echocardiogram Brain natriuretic peptide Comprehensive metabolic panel Troponin
Respiratory	Signs of respiratory distress Dyspnea with exertion	Unilateral lower extremity swelling Cough or wheezing Increased work of breathing Tachypnea	Pretest probability testing (Wells score, D dimer) Diagnostic imaging Trial of bronchodilator Pulmonary function testing
Gastrointestinal	Nausea/vomiting Feeding intolerance	Diarrhea/constipation Abdominal distension Coughing with feeding	X-ray of abdomen Abdominal ultrasound CT of abdomen Comprehensive metabolic panel Amylase/Lipase
Genitourinary	Pain signs with urination Colicky abdominal pain Pain around menstruation Malodorous urine	Urinary retention Abdominal distension Hematuria	Ultrasound of kidneys and bladder Urinalysis Basic metabolic panel
Musculoskeletal	Pain with movement of joints or extremities Pain with repositioning Prolonged use of anticonvulsants, steroids, or proton pump inhibitors	Joint swelling or deformity Bruising	X-ray of affected joint or extremity Bone density scan

Cervical spine stenosis, causing spinal cord compression, may lead to changes in mobility, strength, and incontinence (1). These outcomes disproportionately affect adults with cerebral palsy and other neurologic syndromes and may have irreversible consequences if not identified early (1). Adults with Down Syndrome are also at risk for cervical myelopathy from atlanto-axial instability (25). Adults presenting with rapid decline in mobility, gait changes, new weakness below the C4–C5 level, or new onset incontinence should be evaluated with x-ray and CT or MRI of the spine (1).

Cardiovascular disease, including coronary artery disease and congestive heart failure, affects a higher prevalence of adults with cerebral palsy than other adults (24). Adults with cerebral palsy are also three times more likely to die from cardiovascular disease (23). Further workup for cardiac sources of pain is indicated, especially for adults with exertional pain, change in exercise capacity, tachypnea, orthopnea, lower extremity swelling, rapid weight gain, and personal or family history of atherosclerosis, hyperlipidemia, or diabetes (22–24). Diagnostic testing may include electrocardiogram, echocardiogram, and lab testing, such as brain natriuretic peptide, blood chemistry, and troponin.

Adults with cerebral palsy also have a higher prevalence of diseases affecting a person's respiratory status, including asthma (24). It is unclear if there is an increased prevalence of venous thromboembolism in adults with CP, but this might be investigated if there are signs such as increased work of breathing, pain or discomfort with exertion, or unilateral leg swelling. If these signs or symptoms are present, initial workup should include pretest probability testing and diagnostic imaging (24). While asthma also leads to increased respiratory distress, it commonly is associated with wheezing on exam and is evaluated with a chest x-ray and trial of bronchodilators, such as albuterol (24). Pulmonary function testing is helpful if the patient is able to complete it.

Low bone mineral density and osteoporotic fractures are common in children and young adults with CP, Down Syndrome, and other disorders that reduce independent mobility, such as spina bifida (3, 24, 26–30). There should be high suspicion for an occult fracture, particularly if they have reduced mobility or inability to bear weight, prolonged use of anticonvulsants, steroids, or proton pump inhibitors, and may have bruising or swelling noted on physical examination (3, 24). Initial imaging includes x-ray of the affected area, as well as a bone density scan.

Osteoarthritis is also more commonly seen in adults with cerebral palsy compared to other adults (3). History will typically describe pain in the person's hands, shoulders, hips, or knees, and is evaluated initially with *x*-ray of the painful joint (3).

## Conclusion

Although pain is both common and associated with a lower quality of life in adults with childhood-onset disabilities, it is frequently underrecognized and undertreated. Additionally, there are no standardized tools for measurement of pain in adults with childhood-onset disabilities or for those with communication difficulties and research is limited.

We propose an approach to pain evaluation that consists of a systematic history, comprehensive physical examination, and judicious use of diagnostic testing to help formulate an appropriate differential diagnosis for adults with childhood-onset disabilities. We also recommend additional consideration of adult-specific diagnoses to include for this population.

In future studies, it will be important to adapt assessment tools for use in adults with childhood-onset disabilities or create new tools with a focus on adult-specific diagnoses. This type of research is vital to improve outcomes such as time-to-diagnosis and accuracy of diagnosis and treatment. By improving these outcomes through increased research, the goal is to provide high-quality, patient-centered, and comprehensive care for all adults with childhood-onset disabilities and communication difficulties.

## Author Contributions

TJ and GN confirm their individual contributions to the paper. Both authors reviewed and approved the submitted manuscript.

## Conflict of Interest

The authors declare that the research was conducted in the absence of any commercial or financial relationships that could be construed as a potential conflict of interest.

## Publisher's Note

All claims expressed in this article are solely those of the authors and do not necessarily represent those of their affiliated organizations, or those of the publisher, the editors and the reviewers. Any product that may be evaluated in this article, or claim that may be made by its manufacturer, is not guaranteed or endorsed by the publisher.

## Footnotes

^1^Originally attributed to Robert M. Bilenker, MD.
